# Investigating discrepancies in demand and access for bariatric surgery across different demographics in the COVID-19 era

**DOI:** 10.1016/j.amsu.2022.104368

**Published:** 2022-08-19

**Authors:** Aashna Mehta, Wireko Andrew Awuah, Jacob Kalmanovich, Helen Huang, Resham Tanna, Duaa Javed Iqbal, Tulika Garg, Halil Ibrahim Bulut, Toufik Abdul-Rahman, Mohammad Mehedi Hasan

**Affiliations:** University of Debrecen-Faculty of Medicine, Debrecen, 4032, Hungary; Sumy State University and Toufik's World Medical Association, Sumy, Ukraine; Drexel University College of Medicine, USA; Royal College of Surgeons in Ireland, University of Medicine and Health Sciences, Dublin, Ireland; Independent Researcher, Chicago, IL, USA; Dow Medical College, Karachi, Pakistan; Government Medical College and Hospital Chandigarh, India; Istanbul University Cerrahpasa, Cerrahpasa School of Medicine, Istanbul, Turkey; Sumy State University and Toufik's World Medical Association, Sumy, Ukraine; Department of Biochemistry and Molecular Biology, Faculty of Life Science, Mawlana Bhashani Science and Technology University, Tangail, Bangladesh

## Abstract

Obesity affects over 650 million adults worldwide and increases the risk of cardiovascular events, diabetes, and hypertension. While lifestyle recommendations are popular management options, bariatric surgery has emerged as a standard of care in refractory cases, reported to cause at least a 30% reduction in mortality. In addition, it mitigates obesity-related complications leading to a significant improvement in the quality of life for morbidly obese patients (BMI >40). Despite the numerous benefits, demand and access to bariatric surgery vary across different demographics such as age, gender, and socioeconomic status. This demand and access were further reduced due to the COVID-19 pandemic. This has resulted in cancellations of elective surgeries such as weight loss procedures and promotes a sedentary lifestyle which has short-term and long-term detrimental consequences on the health of obese patients. In the context of the prevalent epidemiological trends, this reduction in bariatric services will disproportionately affect the elderly, males, low SES, and African Americans. This editorial highlights the prevalent discrepancies in demand and access to bariatric surgery amidst the COVID-19 pandemic, and possible recommendations to improve overall access and utilization of bariatric services in morbidly obese patients belonging to all demographics.

## 1 Introduction

Obesity aff.ects over 650 billion adults, with an additional 167 million people projected to be unhealthy by 2025 due to obesity or being overweight [1,2]. Obesity increases the risk of complications including hypertension, diabetes, and cardiovascular diseases [3]. As defined by the World Health Organization (WHO), with a BMI>30 are categorized as obese [1]. Obesity is classified into three levels: grade I (BMI of 30.0–34.9), grade II (BMI of 35.0–39.9), and grade III (BMI of 40.0) [3]. Obesity prevalence varies across different demographics such as age, gender and it disproportionately affects individuals belonging to low socioeconomic status and underserved communities with fewer resources and support at their disposal [3].

While lifestyle modifications such as diet and exercise play a significant role in the management of obesity, weight-loss procedures such as laparoscopic Roux-en-Y gastric bypass (RYGB) and sleeve gastrectomy have emerged as a standard of care to treat morbid obesity (defined as BMI>40 according to the American Society of Metabolic Diseases) with mortality reductions to up to 30% [[3], [4], [5]]. In addition, bariatric procedures can improve gut microbiota composition, and reduce inflammation, and leptin levels associated with reduced BMI [3].

Despite the beneficial effects of bariatric surgery on the quality of life in obese patients, there are observable differences in demand and access to bariatric surgery in different patient groups. With extensive cancellations for elective procedures such as bariatric surgeries, it is imperative to consider the possible impact on prevailing inequities in access and long-term health outcomes [6]. In the context of the current pandemic and growing prevalence of obesity, this editorial highlights prevalent trends for bariatric surgery access in different demographics, information gaps in current knowledge, and possible recommendations to improve equity and access to essential care.

## 2 Concerns over access to bariatric surgery in different demographics in the COVID-19 pandemic

i. Age

As a result of the pandemic, age-related discrepancies in demand and availability to bariatric surgery may be noticed. According to the Center for Disease Control (CDC), more than 40.9% of American adults delayed getting medical assistance, with 55.2% of those aged 18 to 24 showing higher COVID-19-related anxiety, whereas 65.0 and 65.3% of those aged 65 to 74 and > 75 indicated greater COVID-19-related dread [7,8]. Furthermore, the postponement of procedures added to the patients' worry by forcing them to treat the condition at home [9]. This might result in a cyclical loop in which anxiety disrupts visits, which causes more anxiety.

Furthermore, the inability of older patients to obtain procedures may also contribute to the age-related disparity in avoidance. According to Park et al., during the pandemic Medicare seniors in the United States with more comorbid diseases reported having more difficulty accessing medical care than beneficiaries with fewer comorbidities [10]. The rise in anxiety and reduction in accessibility may explain the age-related disparity in bariatric surgery access; however, more research is needed for a conclusive answer.

ii. Gender

Gender disparities, in addition to age differences, have emerged as a potential contributing reason to the fall in Bariatric Surgery demand and access [4]. Prior to COVID-19, women made up 80% of bariatric surgery patients [11]. This is partly explained by the psychological and cultural constructs, which result in gender disparities in perceptions of body weight. This is supported by a systematic study that demonstrated an association between the female gender and a greater willingness to undergo bariatric surgery [12,13].

Also, a drop in healthcare usage has been observed during the pandemic with visits, hospitalizations, diagnostic procedures, and therapeutic treatments down 42.3%, 28.4%, 31.4%, and 29.6%, respectively [14]. This decrease in healthcare utilization may exacerbate previously identified gender differences in bariatric surgery availability. Furthermore, the International Federation for the Surgery of Obesity and Metabolic Disorders (IFSO) made guidelines suggesting all elective procedures for bariatric surgery be postponed during the pandemic [13]. Only emergency treatments are permitted with more conservative therapy indicated for COVID patients [15]. Given that men are more prone than women to contract COVID-19, this regulation may dissuade men from undergoing bariatric surgery [16]. These circumstances may have led to continued gender disparity in bariatric surgery and increased frequency of obesity-related problems in males [17].

iii. Socioeconomic Status

Social-economic status (SES) has consistently been shown to influence the epidemiology of obesity and access to bariatric surgery [18]. A study concluded that people belonging to poor backgrounds and rural areas had considerably less number of procedures despite the number of indications [19]. The disparity among patients receiving bariatric surgery could be tied to its relatively high expense with the average hospital charging $37,000 for one bariatric surgery procedure [20]. Patients with low SES, also report poorer outcomes and an increased risk of postoperative complications [21].

These can be explained by reasons like economic restrictions and a lack of available services, which were especially emphasized during the COVID-19 epidemic. The causes for the lower number of procedures for persons in low-income regions are often related to restricted access to medical care, poor health prior to care, and a lack of opportunity to obtain treatment from well-known facilities. Furthermore, low-income populations are unable to cover the price of bariatric surgery treatments and report poor postoperative quality of life [21]. This was rendered more difficult during the pandemic, as some researchers hypothesized that the indirect economic impacts of the COVID-19 pandemic harmed patients with lower socioeconomic status due to the difficulty to recoup lost wages from unemployment. According to Geranios et al., community groups with lower SES had the poorest economic results from job losses and were more likely to be impacted by layoffs as a result of the pandemic [22]. Delayed care has been found among low-income patients due to a lack of health insurance and a lack of healthcare infrastructure in the areas [23]. The increase in unemployment, among other financial insecurities caused by the COVID-19 epidemic, is likely to have led to the loss of employer-provided health insurance, which disproportionately impacts low-income households without general coverage [24].

ii. Race and Ethnicities

Race has also been observed as a possible determinant of patient access and outcome post-bariatric surgery during the COVID-19 pandemic. Prior to the pandemic studies exploring obesity prevalence in different racial backgrounds demonstrate that African-American women are at the most risk followed by Hispanic women [25]. Factors contributing to obesity in certain races include genetics, diet, physical activity, psychological stress, income, and discrimination [[25], [26], [27]]. Similarly, Latinos/Hispanics have a low rate of bariatric surgery which is attributed to inequality in access to care and financial coverage, low referral rates by primary care physicians, and societal values about obesity combined with distrust of the healthcare system [28,29].

This disparity of access based on race could be exacerbated during the COVID-19 pandemic. Increased psychological stress as a result of the pandemic is leading to maladaptive eating habits, potentially increasing the risk of obesity, especially in racial and ethnic minorities due to limited resources [30]. Subsequently, resulting in disproportionate avoidance of healthcare services as well as worsening emotional eating and binge-eating symptoms before or after surgery leading to worse outcomes [31]. This is one way that the COVID-19 pandemic increased the racial disparity seen in access, demand, and results of bariatric surgery.

Other than fear, financial hardship has been seen to disproportionately affect racial minorities. As the pandemic continues to be contained by vaccines, work has resumed, and black patients are seen to be working multiple jobs in comparison to white patients, whose average pay is insufficient to cover their insurance [31]. This can exacerbate socio-economic divisions, leading to increased obesity prevalence and greater racial disparities in access to weight loss procedures. Clinical research, reviews, and advocacy are valuable and needed to promote equity in access to bariatric surgery across different races and ethnicities.

## 3 Challenges and information gaps

Numerous challenges have surfaced in light of the COVID-19 pandemic that may play a role in the decreased demand and access to bariatric surgery. These challenges include the reduction and postponement of medical care, increased anxiety, unemployment, financial instability, and lack of information on current epidemiological trends.

Due to the pandemic, a drop in healthcare usage and a cancellation of surgeries were noted. In fact, all elective surgeries were notified to be postponed by most surgical societies with bariatric surgery being one of the first affected which creates a delay in proper care [[31], [32], [33], [34]]. The rationale behind this is the increased risk of contracting COVID-19 infection and complications (higher ICU stay and oxygen requirement) in patients with obesity or other comorbidities including metabolic syndrome as well as an increased risk of mortality [34]. The drop in healthcare usage can also be attributed to the increased anxiety associated with the pandemic which discourages hospital and clinic visits [15].

Another issue exacerbated by the pandemic is increasing unemployment and financial insecurity. Unemployment in countries such as the United States peaks at 14.8%, leaving many individuals without financial support [35]. This has an impact on healthcare since it limits the sorts of therapy available, particularly for people with lower socioeconomic status and those with insurance issues. As a result, some people lack the financial wherewithal to pay for medical operations such as bariatric surgery [19].

There is a relative scarcity of recent data on obesity prevalence in the pandemic, as well as, the influence of the pandemic to access to bariatric surgery in different demographics (age, gender, SES, race, and ethnicities) which creates a knowledge deficit in our understanding of bariatric care access. It is concerning considering the cancellations of bariatric surgeries (estimated to be much lower than the 250,000 procedures performed in 2019) which could further reduce access to underserved communities, considering the prevalent inequities [6,36].

All these challenges, especially those as a result of resource and knowledge deficit, considerably limit equivalent utilization of bariatric care across different demographics and as such warrant further investigation.

## 4 Recommendations and conclusions

Implementing strategies to improve access and demand for bariatric surgery is paramount. Based on prior literature, low SES and racial minorities, particularly African-Americans and Hispanic populations, are more likely to experience financial instability that directly affects access [37]. At the same time, these communities also experience a higher prevalence of obesity. Obesity care requires frequent pre- and postoperative evaluations to maintain optimal nutrition for patients, which can involve additional costs and travel. Utilizing telehealth for patient consultations, especially in preliminary stages and follow-ups can encourage higher referrals, and improve care access in underserved communities [38,39]. In 2019, telehealth bariatric services were deployed in rural Canada and found to facilitate follow-ups in rural patients. Another systematic review on telehealth use in low SES communities reported efficient consults, improved access to care, and lowered overall healthcare costs (travel and onsite resource expenditure) [38,49].

Another advantage is convenience, consultations can be held remotely so patients can maintain work and family obligations, particularly in low SES communities, who are likely to work multiple jobs to make financial ends meet. Telehealth also mitigates COVID-19-related anxiety more often observed in the elderly and males, who are more likely to contract COVID-19 and associated complications. Therefore, remote consultations can reduce the risk of transmission in these demographics and encourage compliance with consults. Though e-health illiteracy can be argued as a possible limitation to telehealth utilization, it is generally reported at very low rates and is a common misconception of rural inaccessibility.

Further evidence-based, multicenter cohort studies could establish the epidemiological trends in demand and access to bariatric services in different demographics and their relationship with obesity prevalence. Thereby, providing insightful information to strategize health policies that can improve access in high-risk groups (elderly, males, low SES).

Additionally, government and public health policy should be updated to provide resources and funding for research on the effectiveness of telehealth compared to traditional on-site services in rural, underserved communities.

**Fig. 1:**
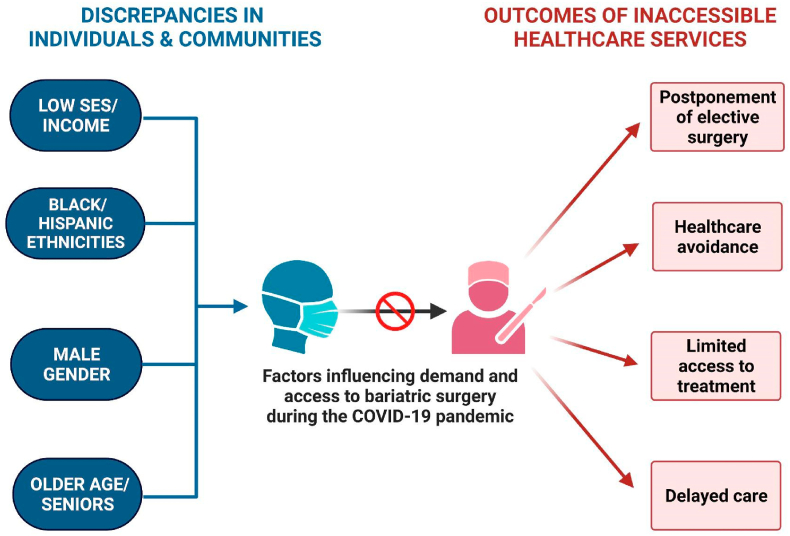
Schematic representation of factors associated with reduced bariatric surgery demand and access during the COVID-19 Pandemic.

Incorporating epidemiological trends affecting bariatric surgery into medical education and decision-making discussions with patients may aid in raising awareness of these disparities and help mitigate the impact of these factors on access and can significantly improve the prognosis and quality of life in obese patients from all demographics (Fig. 1).

## Ethical approval

N/A.

## Sources of funding

N/A.

## Author contribution

Contribution to the work's conception and design: All authors under the supervision of Aashna Mehta, Wireko Andrew Awuah, and Mohammad Mehedi Hasan. All authors worked together to draft the work and revise it critically, with the help of Aashna Mehta, Wireko Andrew Awuah, and Mohammad Mehedi Hasan. The final version of the manuscript was read and approved by all of the authors.

## Conflicts of interest

N/A.

## Consent

N/A.

## Registration of research studies

1. Name of the registry: N/A
2. Unique Identifying number or registration ID: N/A
3. Hyperlink to your specific registration (must be publicly accessible and will be checked): N/A

## Guarantor

Mohammad MehediHasan Department of Biochemistry and Molecular Biology, Faculty of Life Science, Mawlana Bhashani Science and Technology University, Tangail, 1902, Bangladesh. Email: mehedi.bmb.mbstu@gmail.com.

## Data availability statement

No data available.
